# Children's Facial Trustworthiness Judgments: Agreement and Relationship with Facial Attractiveness

**DOI:** 10.3389/fpsyg.2016.00499

**Published:** 2016-04-12

**Authors:** Fengling Ma, Fen Xu, Xianming Luo

**Affiliations:** ^1^Department of Psychology, Zhejiang Sci-Tech UniversityHangzhou, China; ^2^School of Environmental Science and Public Health, Wenzhou Medical UniversityWenzhou, China

**Keywords:** trustworthiness judgment, attractiveness, face, agreement, school-aged children

## Abstract

This study examined developmental changes in children's abilities to make trustworthiness judgments based on faces and the relationship between a child's perception of trustworthiness and facial attractiveness. One hundred and one 8-, 10-, and 12-year-olds, along with 37 undergraduates, were asked to judge the trustworthiness of 200 faces. Next, they issued facial attractiveness judgments. The results indicated that children made consistent trustworthiness and attractiveness judgments based on facial appearance, but with-adult and within-age agreement levels of facial judgments increased with age. Additionally, the agreement levels of judgments made by girls were higher than those by boys. Furthermore, the relationship between trustworthiness and attractiveness judgments increased with age, and the relationship between two judgments made by girls was closer than those by boys. These findings suggest that face-based trait judgment ability develops throughout childhood and that, like adults, children may use facial attractiveness as a heuristic cue that signals a stranger's trustworthiness.

## Introduction

People often form first impressions about others based on their physical appearance—they judge the book by its cover. This strategy serves as an efficient and effective strategy to make interpersonal decisions during social interactions when other information is lacking (Todorov et al., 2005; Bar et al., 2006; Willis and Todorov, 2006; Ballew and Todorov, 2007). One commonly made social decision is a trustworthiness judgment.

The literature has indicated that adults use each other's facial appearances to make trustworthiness judgments just 50 ms after seeing another person's face (Willis and Todorov, 2006; Engell et al., 2007; Oosterhof and Todorov, 2008; Todorov et al., 2009), and that amygdala activity increases with the degree of facial untrustworthiness (Winston et al., 2002; Oosterhof and Todorov, 2008; Todorov and Engell, 2008). Furthermore, face-based trustworthiness judgments are high in agreement among adults (Oosterhof and Todorov, 2008; Todorov et al., 2008). This agreement occurs even when the faces in question belong to a race with which adults are entirely unfamiliar (Xu et al., 2012; Birkás et al., 2014). A recent study reports that older and younger adults displayed similar levels of within-age agreement in their impressions of trustworthiness (Zebrowitz et al., 2013). These findings suggest that adults may rely on certain common facial properties to make trustworthiness judgments (Todorov et al., 2008). Existing work has consistently supported the close relationship between facial trustworthiness and attractiveness judgments (Willis and Todorov, 2006; Oosterhof and Todorov, 2008; Xu et al., 2012). Moreover, Todorov et al. (2009) found that adults indeed perceived more attractive faces to be more trustworthy.

Although a growing body of evidence suggests that adults have evolved into highly sophisticated decoders of trustworthiness based on facial properties alone (Winston et al., 2002; Engell et al., 2007), little research has focused on the development of facial trustworthiness judgment during childhood. Nevertheless, the evidence has demonstrated that children can make social trait judgments (e.g., trustworthiness, dominance, competence, aggressiveness, and attractiveness) based on facial appearances (Keating and Bai, 1986; Antonakis and Dalgas, 2009; Vannatta et al., 2009; Short et al., 2012; Caulfield et al., 2014; Cogsdill and Banaji, 2015; Ewing et al., 2015; Ma et al., 2015). For example, Cogsdill and Banaji (2015) find that 3- to 12-year-old children selected “nice/mean” novel faces in a manner similar to adults. Using the Token Quest paradigm, Ewing et al. (2015) investigate the influence of facial appearances on trust behavior across development. They observed that, like adults, 5- to 10-year-old children selectively invested tokens in trustworthy-looking partners rather than untrustworthy-looking partners. In developmental psychology, most facial trait judgments concentrate on attractiveness. Extensive evidence suggests that from an early age, children exhibit clear preferences for adult-judged attractive vs. unattractive faces (Langlois et al., 1987; Slater et al., 1998; Ramsey et al., 2004). Children also use perceptions of physical attractiveness to make decisions in their social interactions (Cross and Cross, 1971; Dion, 1973; Langlois et al., 1990; Slater et al., 1998; Vannatta et al., 2009; Bascandziev and Harris, 2014).

The ability to infer social traits is a crucial component of social functioning and development. Previous studies have focused primarily on the inference of social traits from facial appearances in infancy and early childhood; little is known about school-aged children. Therefore, we do not know whether the ability to infer social traits based on face improves continuously during childhood. Furthermore, compared with preschoolers, school-aged children are gradually developing independence from their parents and have more opportunities for independent participation in social activities. In fact, we also believed that school-aged children may have some experience similar to judging strangers' trustworthiness from faces. For example, when children get lost, they choose whom to ask the way. The ability to infer trustworthiness based on facial appearances during initial interactions is important for self-protection, social adjustment, and the preservation of wellbeing (Rotenberg et al., 2004, 2005).

Thus, this study aimed to explore the developmental changes in 8- to 12-year-old children's trustworthiness judgments of novel faces. Specifically, we were interested in two questions. The first concerned whether children's trustworthiness judgments based on novel faces were consistent with those of adults (with-adult agreement) and with those of individuals their own age (within-age agreement), and their age-related differences. If children could make consistent judgments, then the second question further explored whether like adults, children used facial attractiveness as a shortcut or heuristics cue for trustworthiness judgments and their age-related differences.

For the first question, we expected that children's trustworthiness judgments of novel faces were similar to those by adults and by individuals their own age. Existing evidence proves that preschoolers can distinguish trustworthy-looking faces from untrustworthy-looking faces in the same pattern as adults (Caulfield et al., 2014; Cogsdill et al., 2014; Ewing et al., 2015). However, beyond the similar preferences between two extreme faces, we expected age-related differences in the agreement levels of facial judgment during childhood. Consistent with this expectation, evidence showed that although children could make consensus nice or mean judgments in the same manner as adults, this consensus (with-adult agreement) increased with age during ages 3 to 12 (Cogsdill and Banaji, 2015). Similarly, previous studies report that there are developmental changes in the perception of facial attractiveness after infancy and even until puberty (Geldart et al., 1999; Cooper et al., 2006). One possibility is that due to limited face perception ability, there are age variations in discrimination between subtle facial differences during development (Bruce et al., 2000; Mondloch et al., 2003, 2010). Another possibility is that, according to an experience-based explanation (Geldart et al., 1999; Cooper et al., 2006), due to limited face experience, children could not form adult-like trustworthy-looking prototypes. Young children's trust originates from close caregivers, and different individuals have different (and different-looking) significant others, friends, and foes. Therefore, one's face experience most likely influences his/her judgments of novel faces (Smith and DeCoster, 2000; DeBruine, 2002). Given the above evidence, we assumed that agreement levels in children might be weaker than those in adults.

Regarding the second question, existing research finds that trustworthiness judgment is closely associated with facial attractiveness (Buckingham et al., 2006; Willis and Todorov, 2006; Oosterhof and Todorov, 2008; Todorov et al., 2009; Xu et al., 2012). If children's judgment patterns were similar to those of adults, then we expected that children's trustworthiness judgments of novel faces were also related to attractiveness. The “beauty is good” stereotype provides other possible support for this hypothesis. The beauty halo effect leads to systematic human perceptual biases, and considerable research has supported that people with more attractive faces are judged more positively on a host of personality traits (Eagly et al., 1991; Langlois et al., 2000; Zebrowitz and Montepare, 2008). Furthermore, this stereotype emerges early in life (Dion et al., 1972; Langlois and Stephan, 1977; Eagly et al., 1991; Langlois et al., 2000; Ramsey et al., 2004; Bascandziev and Harris, 2014). Dion reported that preschoolers are drawn to attractive children as potential friends and that they exhibit a corresponding dislike for unattractive children (Dion, 1973). Bascandziev and Harris also reported that preschoolers preferred to seek and accept information from more attractive people (Bascandziev and Harris, 2014). During initial interaction, we have little information and time to learn about a novel individual's ability, honesty, and benevolence to make a reliable decision about his/her trustworthiness. Thus, we assumed that like adults, children may also use facial attractiveness—the readily accessible and useful indicator of social dispositions—as a heuristic cue for signaling a stranger's trustworthiness.

In this study, we used a data-driven statistical model of 3D faces to generate 200 adult male faces with neutral expressions. Children ages 8 to 12 and a comparison group of adults were asked to judge the faces' trustworthiness and attractiveness. The with-adult and within-age agreement of facial judgments and their relationships to facial attractiveness were analyzed.

## Methods

### Participants

The total valid sample consisted of 138 participants, including 34 8-year-olds (*M* = 8 years, *SD* = 4 months; 17 boys), 34 10-year-olds (*M* = 10 years, *SD* = 5 months; 17 boys), and 33 12-year-olds (*M* = 12 years, *SD* = 4 months; 17 boys), 37 undergraduates were recruited as a comparison group (*M* = 20 years, *SD* = 19 months; 16 males). Additional 21 participants were not included in the final analyses for the following reasons: nine participants (including three 8-year-olds, two 10-year-olds, and four12-year-olds) did not understand the task or were not serious; 12 participants (including four 8-year-olds, four 10-year-olds, two 12-year-olds, and two adult) did not perform the attractiveness judgment in the second test. The participants were all Chinese, and most of the sample (92%) was Han Chinese. They were from families of mixed socioeconomic backgrounds.

This study was approved by the local ethics committees [Institutional Review Board (IRB)] of the Zhejiang Sci-Tech University. Written informed consent was obtained from the parents and teachers of all the children included in this study.

### Face stimuli

**Figure 1:**
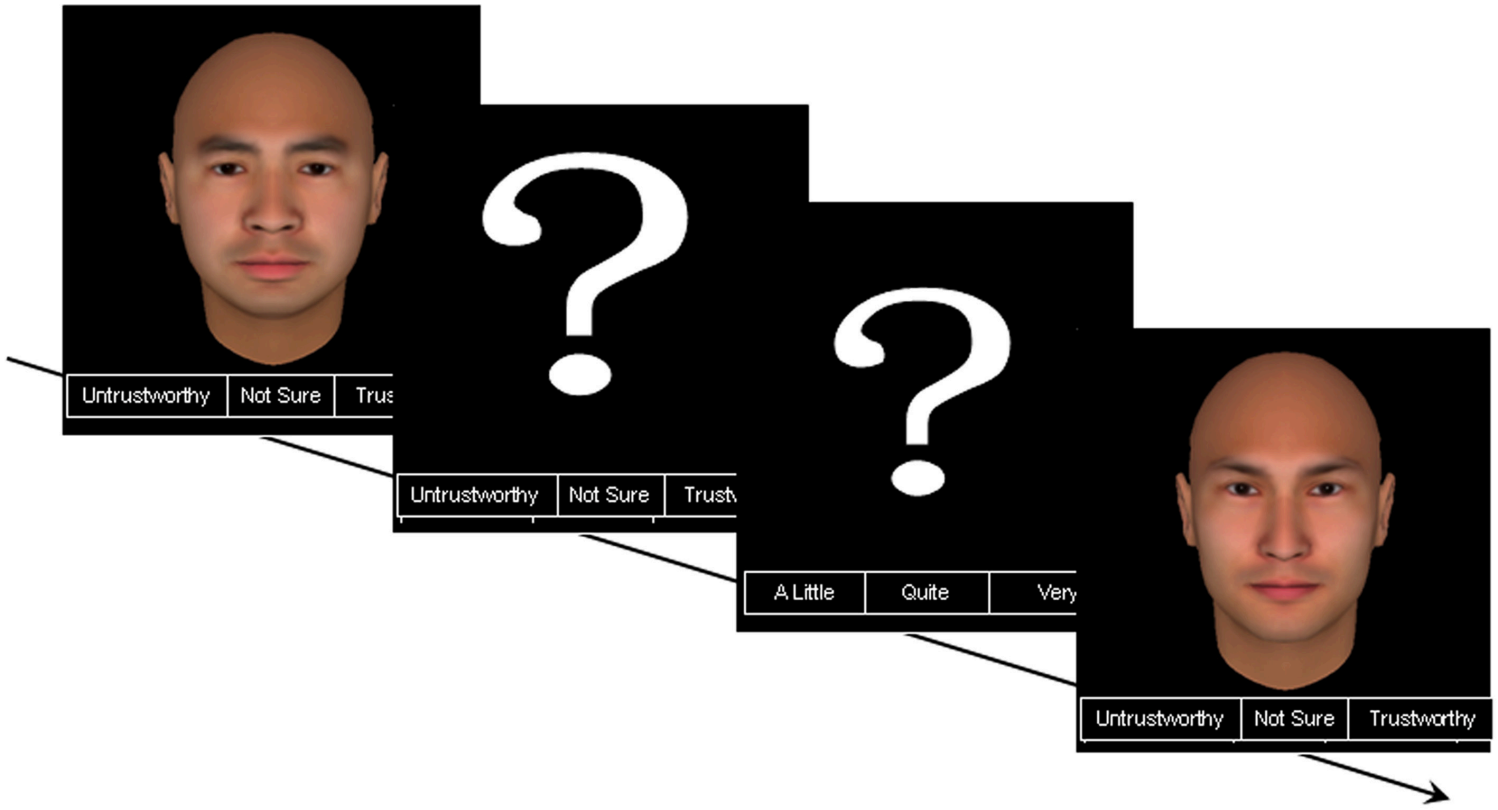
**An example of two trustworthiness judgment trials**.

FaceGen Modeler 3.1 (http://facegen.com) was used to generate emotionally neutral faces with direct gazes. In this study, the 200 randomly generated adult male faces were all East Asian to avoid cross-race effects. The faces were set to appear to be between 20 and 30 years of age. To avoid the influence of symmetry on trustworthiness and attractiveness judgments, all the faces were set to be symmetrical. These procedures resulted in 200 bitmap face images with a resolution of 400 × 400 pixels (see Figure 1 for an example).

### Procedure

The participants were seated in comfortable chairs, and each faced a 14-inch computer screen. E-Prime software was used to individually display the faces in the middle of the screen, which had a black background. The participants were told that they would be shown a series of faces and that their task was to judge how trustworthy (Session 1) and attractive (Session 2) each face was according to their first impressions. They were told that they would make their decision using a button box. The presentation order of the faces was completely randomized. To avoid participant fatigue, four rest intervals were implemented during the testing. The length of the rest intervals was controlled by the children themselves. After resting well, the children pressed the space key on the keyboard to continue the test procedure.

#### Session 1: Facial trustworthiness judgment

Given the limitations of children's cognitive abilities, the rating procedure was simplified as follows: First, the participants were asked to judge whether the face was trustworthy using a 3-point scale labeled “*untrustworthy / not sure / trustworthy*” (or “*trustworthy / not sure / untrustworthy*,” counterbalanced among participants, and the “*not sure*” label was interpreted as “*neither untrustworthy nor trustworthy*”). If the participants rated the face “*untrustworthy*” or “*trustworthy*,” then they were asked to rate the degree of untrustworthiness or trustworthiness on a different 3-point scale labeled “*a little /quite / very*” (or “*very / quite /a little*,” counterbalanced among participants). Thus, the rating scale ranged from –3 (“*very untrustworthy*”) to 3 (“*very trustworthy*”), with 0 indicating “*neither untrustworthy nor trustworthy*.”

Each face was presented in the middle of the screen for 3000 ms (see Figure 1). The rating scale appeared under the face image. There was no time limit for the trials; a trial was terminated when the participant made a choice by pressing the preset buttons on the keyboard. When a face was initially rated “*untrustworthy*” or “*trustworthy*,” a second screen with a question mark appeared, and the participants rated the degree of the face's trustworthiness or untrustworthiness. However, if the participants were “*not sure*” about the face's trustworthiness (or lack thereof), the trial ended immediately, and the participants judged the next face.

First, six practice trials were conducted with novel faces that were not shown during the formal test. All the participants responded systematically during the practice trials and demonstrated that they understood the task. Then, the experimental trials began. The entire rating process lasted approximately 20 min.

#### Session 2: Facial attractiveness judgment

The procedure for the attractiveness judgment was similar to that used for the trustworthiness judgment. The participants were instructed to judge facial attractiveness using a 3-point scale with “*unattractive / not sure / attractive*” (or “*attractive / not sure / unattractive*,” counterbalanced among participants, and the label “*not sure*” was interpreted as “*neither unattractive nor attractive*”); then, the participants continued to rate the degree of unattractiveness or attractiveness on a different 3-point scale with “*a little / quite / very*” (or “*very / quite / a little*,” counterbalanced among participants). Thus, combining the two refined scales into a single overall rating scale created a score that ranged from –3 (“*very unattractive*”) to 3 (“*very attractive*”), with 0 indicating “*neither unattractive nor attractive*.”

All the participants completed the trustworthiness judgments in the first session and the attractiveness judgments in the second session to avoid the influence of the “beauty-is-good” stereotype. In addition, to avoid the possible influence of trustworthiness judgments on attractiveness judgments, all the participants completed the two sessions at intervals of 1 month or more.

## Results

Within each age group, we computed the inter-face reliabilities for trustworthiness and attractiveness judgments for children and adults separately. *Cronbach's* α*lphas* ranged from 0.94 to 0.97, indicating that both children and adults showed high reliability in their judgments.

### Agreement in facial judgments

#### With-adult agreement in facial judgments

To test whether children's trustworthiness and attractiveness judgments were similar to those of adults, we separately correlated each child's ratings of trustworthiness and attractiveness with the mean of all adults' ratings. The correlations for each child were Fisher Z-transformed to normalize them (Franklin and Adams, 2009; Zebrowitz et al., 2013). These correlations provided indices of with-adult agreement for each participant (see Table 1). *T*-tests (comparing means with zero) indicated that all with-adult agreement means were significantly greater than zero (*p* < 0.001), suggesting that overall patterns of trustworthiness and attractiveness judgments among child participants were similar to those among adults. To examine the with-adult agreement differences in the facial judgment type, age, and gender, a 2 (judgment type: trustworthiness, attractiveness) × 2 (gender: boy, girl) × 3 (age group: 8-year-olds, 10-year-olds, 12-year-olds) repeated-measures ANOVA was performed, with judgment type as within-subject variable. The results showed a main effect of judgment type, *F*_(1, 95)_ = 15.54, *p* < 0.001, partial η^2^ = 0.14, suggesting with-adult agreement of attractiveness judgment being significantly higher than that of trustworthiness judgment (for trustworthiness, *M* = 0.18, *SD* = 0.13; for attractiveness, *M* = 0.24, *SD* = 0.15). The main effect of gender was significant, *F*_(1, 95)_ = 9.94, *p* < 0.01, partial η^2^ = 0.10, suggesting that with-adult agreement of girls was significantly higher than that of boys (for girls, *M* = 0.26, *SD* = 0.16; for boys, *M* = 0.18, *SD* = 0.13). Additionally, the age-related differences were significant, *F*_(2, 95)_ = 5.67, *p* < 0.01, partial η^2^ = 0.11. *Post hoc* (LSD) tests indicated that the agreement for facial judgment in the 8-year-old group was significantly lower than that in the 10- (*p* < 0.05) and 12-year-old groups (*p* < 0.01; for the 8-year-old group, *M* = 0.16, *SD* = 0.13; for the 10-year-old group, *M* = 0.22, *SD* = 0.14; for the 12-year-old group, *M* = 0.25, *SD* = 0.16). No significant interactions were found.

**Table 1:** **Descriptive statistics for the with-adult agreements (Z-score) for facial trustworthiness and attractiveness judgments in each group of children (***M*** ± ***SD***)**.

	**8-year-olds (*n* = 34)**	**10-year-olds (*n* = 34)**	**12-year-olds (*n* = 33)**
Trustworthiness judgment	0.14 ± 0.12	0.22 ± 0.14	0.22 ± 0.14
Attractiveness judgment	0.19 ± 0.14	0.25 ± 0.15	0.30 ± 0.17

#### Within-age agreement in facial judgments

To examine the children's and adults' agreement with participants in the same age group in judging trustworthiness and attractiveness, we correlated each participant's ratings with the mean of all the other individuals in his/her age group, excluding the participant. The correlations for each child were Fisher Z-transformed to normalize them (Franklin and Adams, 2009; Zebrowitz et al., 2013, Table 2). These correlations provided indices of within-age agreement for each participant. *T*-tests (comparing means with zero) indicated that all within-age agreement means were significantly greater than zero (*p* < 0.001), suggesting that the overall pattern of judgment in each participant was similar to that of his/her age-mates. To examine the within-age agreement differences in the facial judgment type, age, and gender, a 2 (judgment type: trustworthiness, attractiveness) × 2 (gender: boy, girl) × 3 (age group: 8-year-olds, 10-year-olds, 12-year-olds) repeated-measures ANOVA was performed, with judgment type as within-subject variable. The results showed a main effect of judgment type, *F*_(1, 130)_ = 42.21, *p* < 0.001, partial η^2^ = 0.25, suggesting that within-age agreement of attractiveness judgment was significantly higher than that of trustworthiness judgment (for trustworthiness, *M* = 0.24, *SD* = 0.17; for attractiveness, *M* = 0.33, *SD* = 0.22). The main effect of gender was significant, *F*_(1, 130)_ = 16.05, *p* < 0.001, partial η^2^ = 0.11, suggesting that within-age agreement of girls was significantly higher than that of boys (for girls, *M* = 0.33, *SD* = 0.22; for boys, *M* = 0.24, *SD* = 0.18). The age-related differences were significant, *F*_(3, 130)_ = 78.59, *p* < 0.001, partial η^2^ = 0.66. *Post hoc* (LSD) tests revealed that the within-age agreements in the three child groups were significantly lower than those of the adult group (*p* < 0.001), and within-age agreement in the 8-year-old group was significantly lower than those of the 10- and 12-year-old groups (*p* < 0.05). Additionally, there was a significant two-way interaction between judgment type and age group, *F*_(3, 130)_ = 5.11, *p* < 0.01, partial η^2^ = 0.11. Simple effect analysis revealed that only in the 10-year-old group was there no significant difference in the within-age agreements between trustworthiness and attractiveness judgments; in the other three age groups, the within-age agreement of attractiveness was significantly higher than that of trustworthiness.

**Table 2:** **Descriptive statistics for the within-age agreements (Z-score) for facial judgments in each age group (***M*** ± ***SD***)**.

	**8-year-olds (*n* = 34)**	**10-year-olds (*n* = 34)**	**12-year-olds (*n* = 33)**	**Adults (*n* = 37)**
Trustworthiness judgment	0.12 ± 0.09	0.20 ± 0.12	0.19 ± 0.12	0.44 ± 0.15
Attractiveness judgment	0.19 ± 0.13	0.22 ± 0.14	0.27 ± 0.16	0.60 ± 0.15

### The relationships between facial trustworthiness and attractiveness judgments

Based on the gender effect in the agreement of facial judgments, we separately analyzed the relationship between the two judgments in boys and girls. We averaged the facial judgments of boys and girls to obtain the mean trustworthiness and attractiveness score for each face in each age group. Paired *T*-tests showed that the trustworthiness and attractiveness judgments were significantly different from each other in each age group (*p* < 0.001).

We conducted Pearson correlation analyses to examine whether the facial trustworthiness scores were correlated with the attractiveness scores, with the adults as the comparison group. The results revealed that these two scores were highly correlated with each other in each age group for both boys and girls (*p* < 0.001). We used the Fisher transformation to transform r to Z-scores (Table 3) and then subjected the Z-scores to *Z*-tests. Four *Z*-tests were performed to test the gender effect in each age group. The results indicated that the relationships between the two judgments of girls were significantly closer than those of boys in all the child age groups; however, there was no significant gender effect in the adult group. Then, we computed six *Z*-tests between any two age groups to test the age effect. The results showed that the relationships between trustworthiness and attractiveness in all the child age groups were significantly different from that of the adult group; however, the differences between any two child age groups were not significant (Table 4). The above differences were similar in the boys and girls.

**Table 3:** **The *Zr* transformed from Pearson Correlation (*r*) between trustworthiness and attractiveness judgments in each age group for boys and girls**.

	**8-year-olds**	**10-year-olds**	**12-year-olds**	**Adults**
Boys	0.27	0.34	0.42	0.95
Girls	0.56	0.66	0.63	1.10
*Z* (two-tailed test)	2.89^*^	3.18^*^	2.08^*^	1.49

* *p < 0.05 (two-tailed test)*.

**Table 4:** **The results of ***Z***-tests between any two age groups separately for boys and girls on the correlation of trustworthiness and attractiveness judgments**.

	**Boys**	**Girls**
Differences between 8-year-olds and adults	6.75^*^	5.40^*^
Differences between 10-year-olds and adults	6.05^*^	4.37^*^
Differences between 12-year-olds and adults	5.26^*^	4.66^*^
Differences between 8- and 10-year-olds	0.69	0.99
Differences between 8 and 12-year-olds	1.49	0.69
Differences between 10-year-olds and 12-year-olds	0.79	0.30

* *p < 0.05 (two-tailed test)*.

## Discussion

In this study, we investigated the development of facial trustworthiness judgments of made by 8- to 12-year-old children. The participants were asked to rate trustworthiness and attractiveness based on facial appearances. In general, the findings revealed that school-aged children showed consensus in trustworthiness and attractiveness impressions within their own age groups, and they rated the two impressions in a manner similar to that of adults. Some age-related differences were found: as expected, with-adult and within-age agreements of facial judgments increased with age. We also found that facial attractiveness judgments were more consistent than trustworthiness judgments across all age groups. Additionally, we found that girls made facial judgments more consistently with adults and with individuals their own age than boys. Further exploration of the relationships between facial trustworthiness and attractiveness judgments showed that, like adults, close relationships existed between the two facial judgments during childhood, especially for girls; additionally, some developmental changes existed.

Using the same rating procedure as that used for adults, children aged 8–12 were asked to rate randomly generated novel faces on a revised 7-point Likert scale. Overall, children were able to form similar trustworthiness judgments based on facial appearances across individuals. This finding was consistent with existing evidence (Caulfield et al., 2014; Cogsdill et al., 2014; Cogsdill and Banaji, 2015; Ewing et al., 2015). Prior research that used a forced-choice paradigm found that, as early as preschool age, children could distinguish traits (e.g., trustworthiness or untrustworthiness, competence or incompetence, dominance, or submissiveness) from novel faces in the same way that adults do (Keating and Bai, 1986; Antonakis and Dalgas, 2009; Cogsdill et al., 2014).

As expected, our work provides some evidence that facial trustworthiness judgment abilities gradually improve during childhood. Increasing with age, children's trustworthiness judgments were more consistent with those of adults; additionally, the same developmental difference was found—that the consistency of children's judgments with those of individuals their own age increased with age during childhood; even 12-year-old children did not reach an adult level. One explanation for the developmental changes in trustworthiness judgments may be children's immature face-processing abilities. The research suggests that adult-like expertise in face-based judgments is slow to develop from late childhood through adulthood (Bruce et al., 2000; Mondloch et al., 2003). Furthermore, related brain areas that aid in facial trustworthiness judgments, such as the amygdala, continue to develop throughout late childhood and adolescence and show corresponding functional differences (Giedd et al., 1996; Thomas et al., 2007; Todorov et al., 2008; Baron et al., 2011). Another plausible explanation is based on facial experience. Young children's facial experience comes from close caregivers, and different individuals have different (and different-looking) significant others, friends, and foes. This face environment can influence one's trustworthiness judgments of novel faces (Jones and Hill, 1993; Smith and DeCoster, 2000; Elfenbein and Ambady, 2003; Bronstad and Russell, 2007; Zebrowitz et al., 2012).

With respect to facial attractiveness judgments, similar developmental patterns were found. Children could form consensual attractiveness judgments from novel faces; however, the agreement levels of judgments continued to increase with age. These results were consistent with existing findings, suggesting that children made attractiveness judgments similar to those of adults (Dion, 1973). Our findings provide a more comprehensive overview of the development of attractiveness judgments during childhood.

More importantly, children's agreement levels for attractiveness judgments were higher than those for their trustworthiness judgments. These findings confirmed that the ability to perform facial attractiveness judgments appears early in life, and it can shape children's social decision making. As expected, children's trustworthiness judgments were closely related to facial attractiveness judgments. The results were consistent with existing evidence finding that facial cues signaling trustworthiness overlapped with features that drive attractiveness ratings (Willis and Todorov, 2006; Oosterhof and Todorov, 2008; Todorov et al., 2009; Xu et al., 2012; Ma et al., 2015). For example, Xu et al. (2012) reported that, regardless of their facial experience, Caucasian and Chinese participants used brow ridge (high or low), cheekbones (shallow or pronounced), and face (heavy or light) for facial trustworthiness and attractiveness judgments. Similarly, Ma et al. (2015) also found that children aged 8–12 used the same facial features (such as the shape of the brow ridge, chin and nose) for both trustworthiness and attractiveness judgments in a similar pattern to adults. Another possibility for the close relationship may be that a more attractive face biases people to rate a person as more positive along many dimensions (such as trustworthiness). That is the “beauty is good” stereotype, which appears at a very early age. Even newborn babies display preferences for adult-judged attractive faces, and 1-year-old babies attribute positive behaviors and traits to attractive people and select attractive individuals more than unattractive ones as playmates (Dion, 1973; Langlois et al., 1990; Slater et al., 1998). When encountering unfamiliar individuals, attractiveness may first act as a simple, efficient facial cue for trustworthiness judgments and then induce subsequent approach-or-avoid actions.

We also found that the relationships between trustworthiness and attractiveness judgments in children groups were significantly weaker than that of the adult group. One explanation for this age effect may be that, facial experience and social experience might serve to refine this link between facial attractiveness and trustworthiness. Unlike adults, children participants are more likely to use unique standards (such as faces that resemble their own or the “look” of an important person) to judge facial trustworthiness rather than the shared standards used across other raters. As facial experience increases, common facial cues may play a substantial role in the trustworthiness and attractiveness judgments, as in adults (Cooper et al., 2006). Additionally, consistent with the social learning mechanism, the “beauty is good” impression may gradually develop through children's daily experiences in witnessing the association between attractive individuals and trustworthy behaviors. As Smith and DeCoster proposed, learning about this association takes a long time and requires ample experience (Smith and DeCoster, 2000). Another compounding factor for this age effect may be memory ability differences between children and adults. During the same intervals between facial trustworthiness and attractiveness judgments, adults may have more memory of these stimuli faces, so the degree of correlation between the two judgments was higher than those of children. Further work is needed to verify these findings.

Additional interesting finding was gender effects. Girls made trustworthiness and attractiveness judgments more consistently across individuals in both child and adult groups. Additionally, the relationships between two judgments made by girls were closer than those by boys. These results suggested that girls were more sophisticated at decoding facial trustworthiness and more dependent on facial attractiveness as a heuristic cue to decide who was trustworthy. To our knowledge, no research has reported the stable gender effects on the facial trustworthiness judgment. One plausible explanation was facial trait inference created by variations in facial expression (Zebrowitz and Montepare, 2008), and women have a significant advantage over men in interpreting facial expression information (Judith, 1978). Existing literature has examined the differences in the attractiveness ratings of female and male faces by female and male raters; however, the results were mixed (Wernick and Manaster, 1984; Furnham et al., 2004; Foos and Clark, 2011). Some research proposed that the gender differences in trait judgments may be unstable (Foos and Clark, 2011). In the present study, one possible explanation for the female's advantage may be that all the stimuli were male faces, which might naturally elicit stronger attractiveness judgments from heterosexual females. These gender effects need to be more carefully examined in further research.

Several important and interesting questions were further explored, as follows: First, prior work focused primarily on the consensus or agreement among trustworthiness judgments based on novel faces; little is known about the sources of idiosyncratic variation in facial judgments, particularly in children. For example, adults perceive self-resembling faces as more trustworthy and show more trusting behaviors in economic games with self-resembling patterns (DeBruine, 2002; Krupp et al., 2008). Willis and colleagues have reported that individual differences in trait anxiety are associated with facial trustworthiness judgments (Willis et al., 2013), and facial expressions play an important role in trustworthiness judgments (Willis et al., 2011a,b). An important question for future research concerns the influential factors of trustworthiness judgments based on novel faces during childhood. Second, the gender effect on the trait judgments is another interesting question to explore further. In the present work, we found that women were better at decoding the trustworthy trait based on novel faces than men. From an evolutionary perspective, in social interaction, men may be more likely to value others' competence, while women may be more likely to focus on others' warmth. Then, in the next step, we will investigate the gender effect on other trait judgments (e.g., dominance, competence) based on faces.

This study addressed a developmental question concerning trustworthiness judgments and their relationship with facial attractiveness. Like adults, children as young as 8 years old can make consistent trustworthiness judgments and attractiveness judgments based on facial appearances. Our results clearly demonstrated that age-related differences exist in the trait judgments of faces; that is, the with-adult and within-age agreement levels of facial trustworthiness and facial attractiveness increased with age during childhood. Furthermore, children's trustworthiness judgments were closely related to facial attractiveness, and this relationship increased with age. We also found that girls were more sophisticated at facial judgments than boys. Overall, our findings provide a more comprehensive overview of the development of trustworthiness judgment and add to the recently growing body of work claiming that attractiveness is a universal facial cue for trustworthiness judgments during childhood.

## Author contributions

FM and FX conceived and designed the experiments; FM and XL performed the experiments; FM and XL analyzed the data; and FM, FX, and XL wrote the paper. All the listed authors have approved the final version.

## Funding

This work was supported by the National Natural Science Foundation of China (grant number: 31170996; 31400892).

### Conflict of interest statement

The authors declare that the research was conducted in the absence of any commercial or financial relationships that could be construed as a potential conflict of interest.
